# Blood-based gene signatures associated with therapeutic response to anti-TNF therapy in rheumatoid arthritis: a combined meta-analytical and machine learning approach

**DOI:** 10.1016/j.ero.2025.11.022

**Published:** 2025-12-30

**Authors:** Yoonjung Choi, Taeseong Kim, Hyun Jeong Kim, Daeyoup Lee, Kyung Eun Lee

**Affiliations:** 1 R&D Center, PxPreMED Co., Ltd, Daejeon, Republic of Korea; 2 College of Pharmacy, Chungbuk National University, Cheongju-si, Chungcheongbuk-do, Republic of Korea; 3 Department of Biological Sciences, Korea Advanced Institute of Science and Technology, Daejeon, Republic of Korea

## Abstract

**Objectives:**

Tumour necrosis factor alpha (TNF-α) inhibitors have significantly improved outcomes in rheumatoid arthritis (RA); however, up to 30% to 40% of patients show inadequate response. This highlights the need for predictive biomarkers to guide personalised therapy. Blood transcriptome profiling is a promising strategy for identifying gene signatures linked to treatment response, though prior studies have shown inconsistent results due to technical and biological heterogeneity.

**Methods:**

We performed a comprehensive meta-analysis of baseline blood transcriptome datasets from 8 independent RA cohorts to identify genes consistently upregulated in patients who responded to TNF-α inhibitors. This analysis identified a core set of 39 recurrent genes (the *recurrent gene set*). To evaluate its predictive relevance, we trained machine learning models using 4 gene subsets: the recurrent set, the patent gene set derived from a biomarker patent, the top-ranked gene set based on Cohen’s *d* effect size, and the full gene set. External validation was performed using an independent dataset.

**Results:**

The recurrent gene set showed strong and consistent predictive performance across machine learning models. Notably, several genes overlapping with the patent and top-ranked sets were among the top contributors, supporting their potential value for future diagnostic applications.

**Conclusions:**

Our study demonstrates the feasibility of transcriptome-guided prediction of response to TNF-α inhibitors in RA. The integration of meta-analysis and machine learning provides a strong foundation for the development of precision diagnostic tools to support personalised treatment strategies in clinical practice.

## Graphical Abstract

**Figure:**
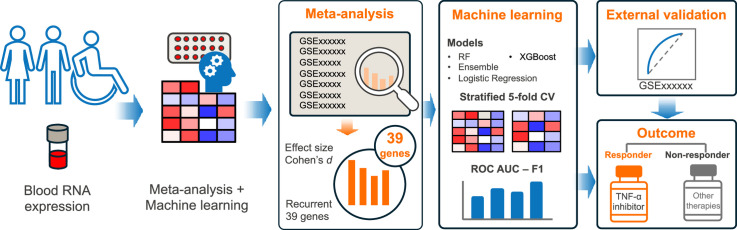

**WHAT IS ALREADY KNOWN ON THIS TOPIC**

• Blood transcriptome profiling has been explored to predict tumour necrosis factor alpha (TNF-α) inhibitor response in rheumatoid arthritis, but results have been inconsistent across studies.

**WHAT THIS STUDY ADDS**

• This study identifies a robust set of recurrently upregulated genes through meta-analysis of 8 blood transcriptome datasets and demonstrates their predictive utility using machine learning models.

**HOW THIS STUDY MIGHT AFFECT RESEARCH, PRACTICE OR POLICY**

• The recurrent gene set may serve as a foundation for developing clinically applicable companion diagnostics to guide TNF-α inhibitor therapy in patients with RA.

Alt-text: Unlabelled box dummy alt text

## INTRODUCTION

Rheumatoid arthritis (RA) is a chronic autoimmune disease characterised by persistent synovial inflammation and progressive joint destruction. When therapeutic interventions fail to adequately suppress the inflammatory process, sustained immune activation can lead to irreversible joint damage, functional impairment, and long-term disability [1].

Over the past 2 decades, the introduction of disease-modifying antirheumatic drugs (DMARDs), including methotrexate (MTX), and biologic DMARDs (bDMARDs) have markedly improved clinical outcomes in RA [[2], [3], [4]]. Among bDMARDs, tumour necrosis factor alpha (TNF-α) inhibitors—such as infliximab, adalimumab, golimumab and etanercept—have demonstrated particularly strong efficacy by targeting key inflammatory pathways. However, despite these therapeutic advances, 30% to 40% of patients fail to achieve an adequate response to TNF-α inhibitors, reflecting the clinical challenge of variable treatment efficacy in real-world settings [[5], [6], [7]]. Biologics, including TNF-α inhibitors, are not only cost-intensive but also carry a risk of adverse effects, further complicating treatment decisions. This highlights the urgent need for predictive biomarkers, especially for TNF-α inhibitors, which are widely used but still lack reliable tools to predict patient response [8,9].

Several transcriptomic studies have since attempted to identify gene expression signatures predictive of anti-TNF-α treatment response [10], but reproducible biomarkers across independent cohorts remain elusive. Although numerous clinical and serological predictors have been explored, none show adequate accuracy for routine practice [11,12]. Advances in high-throughput technologies such as microarray and RNA sequencing now enable comprehensive transcriptomic profiling of blood samples. Because blood is readily accessible and minimally invasive to collect, it serves as a practical source for biomarker development in clinical settings. Multiple studies using blood transcriptome data have reported baseline differential gene expression between responders and nonresponders to TNF-α inhibitors [10,[13], [14], [15], [16], [17], [18], [19]]. However, these findings have often been inconsistent, likely due to variations in study design, cohort size, and transcriptomic platforms, hindering the identification of reproducible signatures [20]. Beyond these inconsistencies, a subset of patients with RA exhibits elevated type I interferon (IFN)-related transcriptional activity. Increased expression of IFN-stimulated genes has been associated with inflammatory burden, disease activity, and differential responses to biologic therapies, including TNF-α inhibitors [21,22]. This IFN signature is one of the most consistently reported molecular features associated with therapeutic response, underscoring the importance of evaluating IFN-related transcriptional patterns in cross-cohort biomarker analyses.

To address this need, meta-analysis of publicly available transcriptome datasets offers a potential solution to these limitations by increasing statistical power and enabling the identification of robust, reproducible gene expression patterns. Nonetheless, cross-cohort meta-analysis of transcriptomic data remains technically challenging due to platform differences, batch effects, and biological heterogeneity. Furthermore, the translation of such findings into clinically useful predictors requires rigorous validation and modelling efforts [23,24].

In this study, we addressed these challenges by performing a comprehensive meta-analysis of baseline blood transcriptomes from 8 independent RA cohorts to identify genes consistently upregulated in responders to TNF-α inhibitors. To evaluate the predictive relevance of the resulting biomarkers, we applied machine learning models as an orthogonal validation approach. We tested the predictive performance of 4 gene subsets: the full gene set, the recurrent gene set, the curated patent gene set, and the top-ranked gene set (based on effect size ranking using Cohen’s *d*). External validation using an independent dataset was conducted to assess the generalisability of the best-performing ensemble model across the 4 gene sets. Our findings highlight the feasibility and promise of transcriptome-guided prediction of drug response in RA, providing a foundation for future development of precision diagnostic tools.

## METHODS

### Sample collection

This study included patients with RA initiating TNF-α inhibitor therapy for the first time (adalimumab, golimumab, or infliximab). Exclusion criteria were age <19 y, prior malignancy, pregnancy or lactation, and incomplete electronic medical records (EMRs). Patients were followed for 6 months after treatment initiation. Recruitment occurred at Ajou University Hospital and Chungbuk National University Hospital between 2017 and 20 December 2019, and clinical data were extracted from institutional EMRs. Collected variables included demographics and baseline clinical characteristics (age, age at diagnosis, sex, body weight, height, rheumatoid factor, anticyclic citrullinated peptide antibody, comorbidities, and comedications). Laboratory values associated with disease activity were obtained at baseline and 6 months, including DAS28-ESR and DAS28-CRP and their components (28 tender joint count, 28 swollen joint count, ESR, CRP, and patient global assessment). DAS28-ESR and DAS28-CRP are composite indices used to monitor rheumatoid arthritis activity, calculated using the 28-joint count along with either the erythrocyte sedimentation rate (ESR) or C-reactive protein (CRP) levels, respectively. Clinical outcomes were defined using European League Against Rheumatism (EULAR) response criteria based on DAS28 [25]. Peripheral blood samples were obtained during routine outpatient visits for subsequent RNA sequencing.

This study was approved by the Institutional Review Boards of Ajou University Hospital (AJIRB-BMR-OBS-17-153) and Chungbuk National University Hospital (2017-06-011-004). All participants provided written informed consent. The study was conducted in accordance with the principles of the Declaration of Helsinki (2013).

### RNA extraction and sequencing

Whole blood samples were collected from RA patients before TNF-α inhibitor treatment and preserved in PAXgene Blood RNA Tubes (PreAnalytiX). Total RNA was extracted according to the manufacturer’s instructions using the PAXgene Blood RNA Kit.

High-quality RNA samples were subjected to DNase I treatment before library preparation. Strand-specific libraries were constructed using the NEXTflex Rapid Directional mRNA-Seq Kit (BIOO Scientific), following the manufacturer’s protocol. Libraries were sequenced on an Illumina NovaSeq platform with paired-end 150 bp (PE150) reads under strand-specific conditions.

### Data preprocessing

For microarray datasets (GSE126476 [15], GSE93272 [18], GSE33377 [10], GSE78068 [19], GSE20690 [17], and GSE12051 [16]), raw expression files were downloaded and normalised using platform-specific pipelines. Probe intensities were summarised at the gene-symbol level after standard background correction and within-array normalisation. For the newly generated RNA-seq dataset (GSE309430), raw FASTQ files were processed through the following pipeline: adapter trimming was performed using Trimmomatic [26], reads were aligned to the human genome (hg19) using STAR v2.4.0 [27], and gene-level quantification was conducted with HOMER [28]. Detailed preprocessing steps are provided in the Supplementary Methods.

### Differential expression analysis

Differential expression analysis was conducted independently for each dataset using appropriate methods for each platform. For RNA-seq datasets, raw count matrices were analysed using the DESeq2 package [29]. For microarray datasets, normalised expression matrices were compared between responders and nonresponders using log₂ fold change estimates. Full analytical procedures and thresholds are provided in the Supplementary Methods.

### Effect size and directional consistency analysis

**Figure 1:**
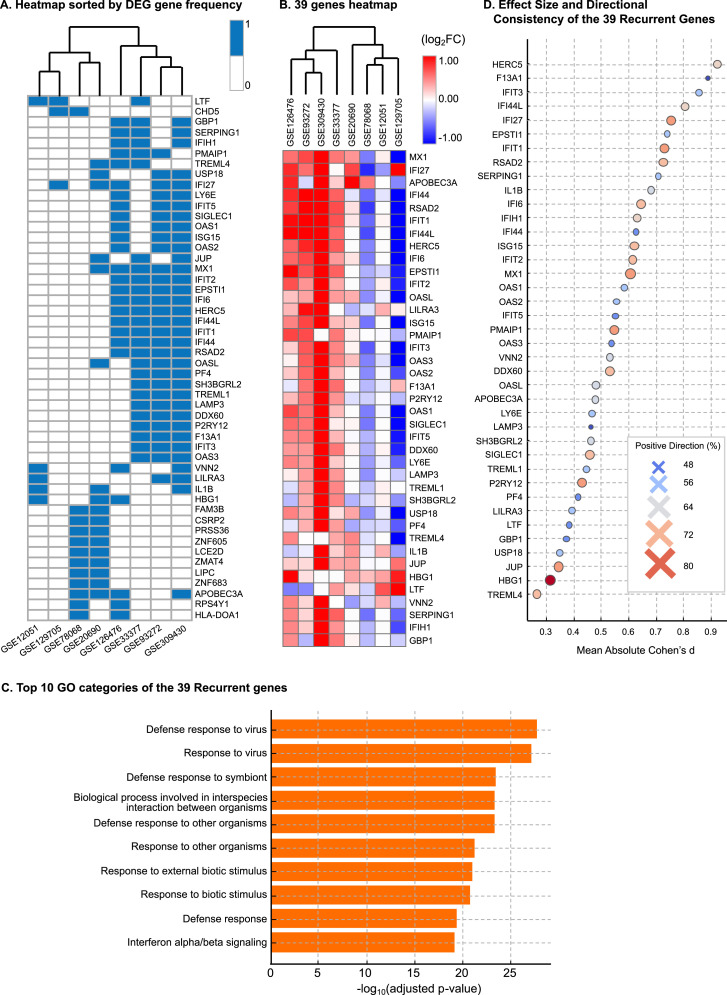
Identification of recurrent predictive gene signatures through blood transcriptome meta-analysis. (A) Binary heatmap indicating whether each gene was identified as upregulated (binary value = 1; blue) or not (binary value = 0; white) in responders compared to nonresponders within each dataset. Gene selection was based on differential expression analysis per dataset. DEG, differentially expressed gene. (B) Hierarchical heatmap of log₂ fold change values for the 39 recurrently upregulated genes. The colour scale reflects relative log₂ fold change in responders vs nonresponders across datasets. FC, fold change. (C) Gene Ontology (GO) enrichment analysis of the 39 recurrent genes. The top 10 significantly enriched biological process terms are shown, ranked by –log₁₀ (adjusted *P* value). (D) Effect size and directional consistency of the 39 recurrent genes. Each dot represents a gene; the x-axis shows the average absolute Cohen’s *d*, while dot size and colour indicate the proportion of datasets in which the gene exhibited a positive effect size (ie, higher expression in responders).

To ensure cross-platform comparability, gene expression values were normalised within each dataset using the most stable housekeeping gene, *PGK1* (phosphoglycerate kinase 1), identified based on low coefficient of variation among 11 candidates (mean CV < 0.03, max CV < 0.06; Supplementary Table S1). CV (coefficient of variation) was used to evaluate the expression stability of the candidate housekeeping genes. Using the *PGK1*-normalised matrices, Cohen’s *d* effect sizes for the 39 recurrent genes were computed for responders vs nonresponders across 8 datasets. For each gene, the mean absolute Cohen’s *d* represented the average effect size, while the proportion of datasets showing positive d values indicated directional consistency. These 2 metrics—mean absolute effect size and directional consistency—were used for ranking and visualising genes in Figure 1D.

### Model training and hyperparameter optimisation

We trained 3 supervised classifiers—Random Forest, XGBoost, and Logistic Regression—together with a soft-voting ensemble. Models were trained using preprocessed expression matrices with responder status as the outcome. Four predefined gene sets were evaluated for comparative prediction performance. Hyperparameters were optimised using stratified 5-fold cross-validation, and the best models were selected based on mean AUC (area under the receiver operating characteristic curve). Detailed model specifications, parameter grids, and implementation code are provided in the Supplementary Methods.

### Model evaluation and statistical validation

Model performance was evaluated using receiver operating characteristic (ROC) curves, AUC, and complementary metrics including accuracy, precision, recall, and F1-score. Generalisation performance was estimated using stratified 5-fold cross-validation, and ensemble predictions were generated through soft voting. Statistical robustness was assessed using permutation testing, in which class labels were randomly shuffled to generate null AUC distributions. Full evaluation procedures and statistical settings are provided in the Supplementary Methods.

### External validation using GSE42296

External validation was conducted using the independent dataset GSE42296 [13], comprising baseline blood transcriptomes from patients with RA treated with TNF-α inhibitors. Expression data were normalised and annotated to gene symbols using standard RMA (robust multi-array average) algorithm for background correction and normalization. The pretrained ensemble classifier (soft voting of Random Forest, XGBoost, and Logistic Regression) with fixed hyperparameters from internal validation was applied without further tuning. Predictive performance was assessed by ROC-AUC and F1-score on the GSE42296 dataset.

## RESULTS

### Recurrent predictive biomarkers from blood transcriptome meta-analysis

We performed a meta-analysis to identify gene expression signatures capable of predicting treatment response to anti-TNFα therapies. Eight independent transcriptome datasets of patients with RA were analysed, comprising 6 public microarray datasets, 1 publicly available RNA-seq dataset, and 1 RNA-seq dataset newly generated from Korean patients with RA in this study (Table). All datasets were derived from blood samples obtained before anti-TNF-α inhibitor treatment, primarily with infliximab. Responders and nonresponders were classified according to the EULAR response criteria, based on DAS28 and CDAI (Clinical Disease Activity Index) scores assessed at 12–48 weeks after treatment initiation [30]. Of the 341 patients with RA, 172 were identified as responders and 169 as nonresponders.

**Table:** Summary of publicly available transcriptomic datasets used in this study

Study ID	Drug	Timepoint (wk)	Baseline DAS28	Response definition	Responders/nonresponders	Response criteria	Platform	Sample source
GSE78068	Infliximab	24	5.3 (4.4-6.2)	CDAI ≤ 2.8 (6 mo)	38/81	CDAI	Agilent 4 × 44K	Whole blood
GSE93272	Infliximab	24	5.24 (SD 1.24)	EULAR good responder (ΔDAS28 ≥ 1.2, DAS28 ≤ 3.2)	10/9	EULAR (DAS28-ESR)	Affymetrix U133 Plus 2.0	Whole blood
GSE20690	Infliximab	14	3.2	EULAR criteria based on DAS28-CRP	38/24	EULAR (DAS28-CRP)	Agilent 4 × 44K	Whole blood
GSE12051	Infliximab	14	3.2	EULAR response based on DAS28	34/7	EULAR	Illumina Sentrix	Whole blood
GSE33377	Infliximab, adalimumab	14	3.2	EULAR response based on DAS28	17/24	EULAR	Affymetrix U133A	Whole blood
GSE126476	Etanercept	48	3.2	EULAR response based on DAS28	12/5	EULAR	Affymetrix U133B	PBMC
GSE42296	infliximab	14	3.2	ACR50/70	6/13	ACR	Affymetrix Human Gene 1.0 ST	PBMC
GSE129705	Infliximab, adalimumab	12	CDAI > 10	EULAR criteria based on DAS28-CRP	18/16	EULAR	Illumina HiSeq 2500	Whole blood
GSE309430	Infliximab	24	3.2	EULAR response based on DAS28	5/3	EULAR	NovaSeq 6000	Whole blood

ACR, American College of Rheumatology; CDAI, Clinical Disease Activity Score; EULAR, European League Against Rheumatism; ESR, Erythrocyte Sedimentation Rate; DAS28-CRP, Disease Activity Score 28 with C-Reactive Protein; PBMC, Peripheral Blood Mononuclear Cell.

To address substantial heterogeneity in platforms and clinical designs, we used a comparison-based meta-analysis strategy that independently processed each dataset rather than integrating raw data [[31], [32], [33]]. DEGs (Differentially Expressed Genes) were identified using platform-specific thresholds: log₂FC ≥ 0.3 for microarray datasets without statistical filters and log₂FC ≥ 1 with nominal *P* < 0.01 for RNA-seq datasets. Relatively relaxed thresholds were applied to capture biologically relevant genes potentially masked by interstudy variability. Only upregulated genes were considered, reflecting the practical utility of elevated expression for diagnostic assay development.

DEG lists from individual datasets were merged, and a binary heatmap was constructed to assess the recurrence of upregulated genes across studies (Fig 1A). This analysis identified a total of 1465 unique genes, from which we selected 39 genes consistently upregulated in at least 3 datasets. Expression patterns of these 39 recurrent genes were visualised using a log₂ fold change heatmap (Fig 1B). Hierarchical clustering (one-minus-Pearson correlation, average linkage) showed concordant upregulation of IFN-stimulated genes such as *MX1, IFI27, RSAD2, IFIT1, IFI44L, ISG15,* and *OAS1*. These genes are well-established targets or downstream effectors of type I IFN and inflammatory signalling pathways known to interact with TNF-α activity [[34], [35], [36]]. GSE78068 and GSE129705 formed a distinct cluster characterised by relative downregulation of these genes, likely reflecting cohort-specific or technical differences.

To examine whether the 39 recurrent genes share meaningful functional characteristics, we conducted Gene Ontology (GO) enrichment analysis. The results revealed significant enrichment in immune-related biological processes, particularly those associated with type I IFN signalling and antiviral defence. Top-ranked GO terms included ‘defense response to virus’ (GO:0051607, adj. *P* = 1.4 × 10^-^²⁸), ‘response to virus’ (GO:0009615), and ‘regulation of cytokine production,’ supporting functional coherence of the recurrent signature (Fig 1C).

To strengthen the meta-analytic rigour and provide a quantitative measure of expression consistency across datasets, we computed Cohen’s *d* effect sizes for all 39 genes [37]. Expression values were normalised in 2 steps. First, raw data were preprocessed using platform-specific methods. Second, we then evaluated the expression stability of 11 commonly used housekeeping genes and identified *PGK1* as the most stable reference across datasets (mean CV < 0.03, max CV < 0.06; Supplementary Table S1). All expression matrices were subsequently scaled relative to *PGK1*, enabling consistent normalisation across diverse platforms. For each gene, the average absolute Cohen’s *d* was used to evaluate effect size magnitude, and the proportion of datasets in which the gene showed a positive effect direction was used to assess directional consistency. These 2 metrics were visualised using a combined dot plot (Fig 1D), and their full numerical values are provided in Supplementary Table S2. Because not all genes were observed across all 8 datasets, directional consistency was calculated relative to the number of datasets in which each gene was present. Notably, several genes, including *HERC5, IFIT3, IFI44L,* and *IFI27*, exhibited both strong effect sizes and high consistency. In addition, KR102429261B1 [38], a patent derived from a single-cohort transcriptome study, also included in our analysis, lists biomarkers such as *IFI44L, IFI27*, and *IFIT1*, which overlapped with our recurrent set. This convergence reinforces the robustness and translational potential of these biomarkers.

Altogether, our meta-analysis identifies a reproducible cross-cohort biomarker signature with strong biological and statistical support, providing a solid foundation for predictive modelling and future clinical application.

### Machine learning-based prediction of TNF-α inhibitor response using transcriptomic features

To evaluate the predictive power of transcriptomic biomarkers for TNF-α inhibitor response in RA, we constructed classification models using 4 distinct gene sets:

(1) the *Full gene set*, consisting of 18,023 genes detected in at least 4 of the 8 transcriptome datasets; (2) the *recurrent gene set*, comprising 39 genes recurrently upregulated across studies as identified through meta-analysis (Fig 1B); (3) the *patent gene set*, consisting of 7 biomarker genes claimed in patent KR102429261B1 (eg, *IFI27, IFI44L*, and *IFIT1*; see Supplementary Table S2); and (4) the *top-ranked gene set*, consisting of 7 genes prioritised based on a 2-metric framework (average absolute Cohen’s *d* and directional consistency) from Figure 1D (eg, *IFI44L, IFIT3*, and *HERC5*; see Supplementary Table S2).

For each gene set, we trained 4 machine learning classifiers—random forest (RF) [39], XGBoost (XGB) [40], logistic regression (LR), and an ensemble model—using stratified 5-fold cross-validation with hyperparameter optimisation via GridSearchCV. Multiple ensemble configurations (RF+XGB, RF+LR, XGB+LR, and RF+XGB+LR) were evaluated, and the RF+XGB+LR combination was selected as the final ensemble model based on its consistently strong performance across all feature sets.

**Figure 2:**
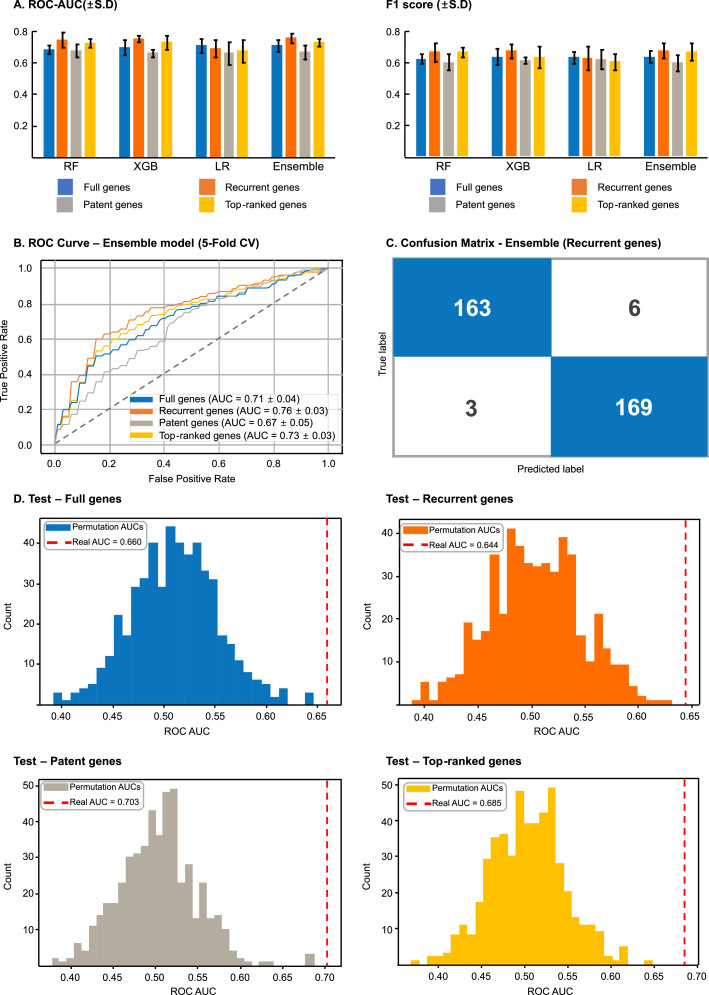
Predictive performance evaluation of gene signatures using machine learning models. (A) Comparison of predictive performance across 4 gene feature sets—full, recurrent (n = 39), patent (n = 7), and top-ranked (n = 7)—using 4 machine learning models: random forest (RF), XGBoost (XGB), logistic regression (LR), and ensemble (RF + XGB + LR). Bar plots show the mean receiver operating characteristic (ROC)-AUC (top) and F1-score (bottom) with SDs from stratified 5-fold cross-validation. AUC, area under the curve. (B) ROC curves of the ensemble model (RF + XGBoost + LR) trained with the best parameters on 4 gene sets: mean AUCs (± SD) were calculated via stratified 5-fold cross-validation. CV, cross-validation. (C) Confusion matrix of the ensemble model (RF + XGBoost + LR) trained with the best-performing parameters on the 39 recurrent gene set. (D) Permutation test results for the ensemble model trained with the best-performing parameters on each of the 4 gene feature sets. Histograms represent the distribution of ROC AUCs from 100 label-shuffled models, and the red dashed line indicates the AUC of the true model.

Model performance was assessed using 5 metrics: ROC-AUC, accuracy, precision, recall, and F1-score (Supplementary Table S3). Bar plots visualising ROC-AUC and F1-score, along with SD error bars, are presented in Figure 2A. The ensemble model (RF+XGB+LR) consistently demonstrated robust performance across gene sets. Notably, the recurrent gene set yielded one of the highest AUC values (0.76 ± 0.03) and the highest F1-score (0.68 ± 0.05), outperforming even the full transcriptome in terms of balance and stability. In addition, both the top-ranked gene set and patent gene set, each comprising only 7 features, achieved reasonably high AUC scores (0.73 and 0.67, respectively) and competitive F1-scores (0.68 and 0.61, respectively). Although the patent gene set underperformed in ROC-AUC, its classification metrics remained consistent, indicating that a subset of its features may contribute useful signals in prediction. Interestingly, the top-ranked gene set, selected based on effect size, performed comparably to the recurrent gene set in terms of F1-score, highlighting the effectiveness of compact, data-driven feature selection strategies. Collectively, these results support the feasibility of using minimal gene sets to achieve high predictive accuracy, thereby facilitating future clinical translation.

To further evaluate classification performance, ROC curves were generated for the ensemble model across all 4 gene sets (Fig 2B). Although Figure 2A quantitatively compared AUC scores, Figure 2B illustrates the model’s behaviour across all classification thresholds. The recurrent gene set exhibited a consistently higher true positive rate across a broad range of false positive rates, reflecting superior discriminative power. Meanwhile, the ROC curves for the compact patent and top-ranked gene sets also remained well above the random baseline, demonstrating reliable predictive signals despite their minimal size. The smooth and well-separated curves across all gene sets support the robustness and generalisability of the ensemble model.

In addition to the threshold-independent evaluation shown in Figure 2B, a confusion matrix was generated for the ensemble model trained on the recurrent gene set using the entire dataset (Fig 2C). The model correctly classified the majority of samples, yielding only 6 false positives and 3 false negatives out of 341 total cases. This result demonstrates a well-balanced trade-off between sensitivity and specificity under a fixed threshold, further supporting the robustness and potential clinical utility of the ensemble model based on a compact, recurrent gene signature. To assess whether the observed predictive performance could be attributed to random chance, we performed permutation testing across all 4 gene sets. In this analysis, sample labels were randomly shuffled 100 times, and models were retrained to establish null distributions of AUC scores. As shown in Figure 2D, for each feature set—including the full, recurrent, patent, and top-ranked gene sets—the AUCs of the true models (red dashed lines) were significantly higher than those obtained from the permuted models (blue histograms). Permutation tests consistently yielded *P* values below 0.01, confirming that the observed classification performance was statistically significant and unlikely to occur by chance under randomised class labels.

Taken together, these findings demonstrate that compact gene sets—particularly those derived through rigorous meta-analysis—can achieve predictive performance comparable to large-scale models.

### External validation and feature prioritisation for clinical translation

To assess the generalisability of our predictive models, we performed an external validation using the publicly available transcriptome dataset GSE42296, which includes peripheral blood gene expression profiles from patients with RA before treatment with infliximab, a TNF-α inhibitor. As this dataset was entirely excluded from model training and parameter optimisation, it served as an appropriate benchmark for independent performance evaluation. We applied the same ensemble classifiers with the best-performing parameters identified in Figure 2 to each feature set.

**Figure 3:**
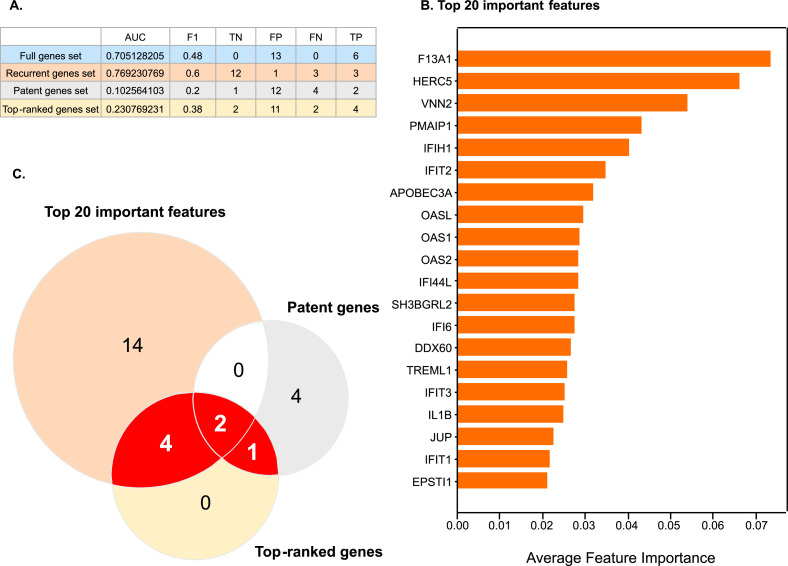
External validation and interpretability of predictive gene sets. (A) Performance of the ensemble model (random forest + XGBoost + logistic regression) on the external validation dataset GSE42296. The model was trained on each of the 4 gene sets using the best-performing parameters. Metrics include AUC (Area Under the Curve), F1-score, and confusion matrix components (TN: true negative, FP: false positive, FN: false negative, TP: true positive). (B) Top 20 most important genes identified by the ensemble model (random forest + XGBoost) trained on the 39 recurrent gene set. Feature importance was calculated as the average of impurity-based importance scores from the 2 tree-based classifiers. (C) Venn diagram illustrating the overlap between the top 20 informative genes (derived from Fig 3B), the patent gene set, and the top-ranked gene set. Two genes, IFI44L and IFIT1, were common to all 3 sets. Notably, 4 genes (F13A1, HERC5, IFIT3, and EPSTI1) were shared between the informative and top-ranked sets, while IFI27 was the only gene overlapping between the patent and top-ranked sets.

As summarised in Figure 3A, the recurrent gene set consistently outperformed the other 3 feature sets, achieving the highest AUC of 0.77 (95% CI: 0.48-0.98) and an F1-score of 0.60 (95% CI: 0.00-0.91) based on 2000 stratified bootstrap resamples. As expected, CIs were wide due to the small sample size, but the directionality of predictive performance was consistent with the internal cross-validation results. To provide additional clinically interpretable performance metrics, we calculated sensitivity, specificity, positive predictive value (PPV), and negative predictive value (NPV) for the recurrent gene-based ensemble model. In the meta-analysis cohort, the model achieved a sensitivity of 0.98, specificity of 0.96, PPV of 0.97, and NPV of 0.98. In the external validation dataset (GSE42296), the same model yielded a sensitivity of 0.50, specificity of 0.92, PPV of 0.75, and NPV of 0.80, indicating that the model maintains high specificity and NPV even in an unseen cohort. These metrics suggest that although the external validation cohort is limited in size, the model may be particularly informative for identifying patients who are unlikely to respond to TNF-α inhibitors. In contrast, the patent and top-ranked gene sets, although they performed well during cross-validation (Fig 2), showed markedly reduced predictive accuracy in the independent validation cohort. These results suggest that models relying on smaller gene panels may be prone to information loss, reducing their ability to generalise to unseen data in real-world settings. To further explore this discrepancy, we computed feature importance scores using the tree-based components-Random Forest and XGBoost-within the ensemble model trained on the recurrent gene set. As shown in Figure 3B, several genes from the patent and top-ranked sets appeared among the top 20 informative genes, indicating that these compact biomarker panels still contain biologically relevant signals. We next examined the overlap between the top 20 informative genes derived from the recurrent gene set, and the patent and top-ranked gene sets (Fig 3C). Two genes—*IFI44L* and *IFIT1*—were consistently shared across all 3 sets, highlighting their central role in predicting TNF-α inhibitor response. Moreover, 6 of the 7 genes in the top-ranked set overlapped with the top 20 informative genes, further underscoring their robustness and potential utility. Despite the limited predictive performance of the smaller gene sets in external validation, the consistent appearance of specific genes across the recurrent, patent, and top-ranked panels reflects their biological relevance. This convergence, which results from independent selection strategies including cross-cohort recurrence, patent curation, and effect size prioritisation, reinforces the robustness of these overlapping genes.

These findings further demonstrate the value of meta-analytic feature selection in identifying stable transcriptomic signals and highlight the importance of integrating such robust features when applying machine learning models to heterogeneous real-world datasets.

## DISCUSSION

In this study, we aimed to identify biomarkers that can predict response to anti-TNF-α therapy in patients with RA through a meta-analysis of blood transcriptome profiles collected at baseline. Despite substantial heterogeneity across cohorts and platforms, we established a robust meta-analytical strategy that enabled the identification of genes consistently upregulated in responders across multiple studies. We further demonstrated the predictive utility of these gene signatures using multiple machine learning models.

Performing a meta-analysis of transcriptome data from multiple independent studies is challenging due to differences in microarray platforms, normalisation procedures, and clinical definitions of response. To address these issues, we implemented a comparison-based strategy that incorporated effect size (Cohen's *d*) and housekeeping gene-based normalisation. This approach proved effective for identifying biomarkers associated with treatment response. Functional enrichment analysis of the 39 gene signatures obtained through this method revealed strong involvement in type I IFN signalling and innate immune pathways, which are well-known to contribute to RA pathogenesis and therapeutic response mechanisms. Moreover, the elevated IFN-stimulated gene expression observed here aligns with prior studies suggesting that baseline IFN activity defines a subset of patients with RA with distinct inflammatory states and variable responses to biologics [21,22]. In contrast, recent work in osteoarthritis reported that a stronger IFN signature predicted treatment refractoriness rather than responsiveness [41] suggesting disease-specific roles of IFN activity. Together, these findings reinforce the relevance of IFN-related transcriptional programs in influencing therapeutic outcomes. An additional consideration is that elevated inflammatory or IFN-driven transcriptional activity may reflect overall immune activation rather than mechanisms specific to TNF-α inhibitor response. Although the recurrent gene signature was derived from anti-TNF-α cohorts and showed consistent predictive directionality in an independent dataset, we cannot exclude the possibility that it captures broader inflammatory states. Future studies comparing different biologic classes, ideally within the same cohort, will be needed to determine the TNF-specificity of these transcriptional predictors.

Beyond these biological insights, both technical and biological heterogeneity warrant consideration. Cohort-level heterogeneity including demographic differences as well as variations in inflammatory burden, disease activity, prior treatments, and sample composition may have introduced molecular noise that diluted drug-specific expression patterns, particularly in analyses combining infliximab- and adalimumab-treated patients. Although our effect-size-based normalisation strategy mitigates some of this variability, it cannot fully eliminate these differences, underscoring the need for future prospective cohorts with standardised sampling and uniform clinical annotation.

Despite this therapeutic and biological heterogeneity, the 39 recurrent gene set achieved the strongest and most consistent performance across all machine learning models, highlighting its reliability and robustness. External validation using the independent GSE42296 cohort further confirmed the generalisability of our transcriptome-based gene signatures for predicting response to anti-TNF-α therapy. Although the external validation cohort included only 19 patients, which resulted in wide CIs, the overall directionality of predictive performance remained consistent with that observed in cross-validation of the training cohorts. These findings suggest that the recurrent IFN-related gene signature retains meaningful predictive value across independent datasets. Although the patent and top-ranked panels did not achieve comparable performance, the substantial overlap of key genes, particularly *IFI44L* and *IFIT1,* with the recurrent set underscores their biological relevance and highlights the potential feasibility of developing more compact biomarker panels for future clinical implementation.

Taken together, our findings provide an important intermediate step toward clinically useful predictors of TNF-α inhibitor response. By integrating heterogeneous datasets and validating recurrent gene signatures across multiple machine learning models as well as in an independent cohort, we establish a scalable framework for treatment-response prediction. Nonetheless, translating these signatures into routine clinical practice will require standardised assay development and evaluation within real-world clinical workflows.

This study highlights the value of transcriptome meta-analysis as a foundation for AI-based drug response prediction. Our end-to-end pipeline, spanning biomarker discovery, algorithmic modelling, and external validation, supports scalable and cost-effective biomarker discovery, with easy applicability to other treatments and diseases. A primary limitation of this study is its reliance on retrospective datasets. Future work will require well-annotated, prospectively collected cohorts to more firmly establish clinical utility. Moving forward, we aim to validate these signatures in a prospective RA cohort and extend our platform to other therapeutic classes, ultimately advancing toward a broader AI-based precision medicine framework for immune-mediated diseases.

## Contributors

YC performed the experiments and carried out transcriptome meta-analysis and machine learning-based data modelling. YC and TK wrote the manuscript. DL and KEL supervised the project.

## Funding

This work was supported by the Basic Science Research Program through the National Research Foundation of Korea (NRF-2020R1F1A1069718) funded by the Ministry of Science, ICT and Future Planning.

## Competing interests

The authors declare no competing interests.

## Appendix Supplementary materials

Image, application 1

Image, application 2

Image, application 3

## Footnotes

Supplementary material associated with this article can be found in the online version at doi:10.1016/j.ero.2025.11.022.
